# Respiratory rehabilitation after acute exacerbation of COPD may reduce risk for readmission and mortality – a systematic review

**DOI:** 10.1186/1465-9921-6-54

**Published:** 2005-06-08

**Authors:** Milo A Puhan, Madlaina Scharplatz, Thierry Troosters, Johann Steurer

**Affiliations:** 1 Horten Centre, University of Zurich, Switzerland; 2 Respiratory Division, Respiratory Rehabilitation, and Faculty of Kinesiology and Movement Sciences, Katholieke Universiteit Leuven, Leuven, Belgium

## Abstract

**Background:**

Acute exacerbations of chronic obstructive pulmonary disease (COPD) represent a major burden for patients and health care systems. Respiratory rehabilitation may improve prognosis in these patients by addressing relevant risk factors for exacerbations such as low exercise capacity. To study whether respiratory rehabilitation after acute exacerbation improves prognosis and health status compared to usual care, we quantified its effects using meta-analyses.

**Methods:**

Systematic review of randomized controlled trials identified by searches in six electronic databases, contacts with experts, hand-searches of bibliographies of included studies and conference proceedings. We included randomized trials comparing the effect of respiratory rehabilitation and usual care on hospital admissions, health-related quality of life (HRQL), exercise capacity and mortality in COPD patients after acute exacerbation. Two reviewers independently selected relevant studies, extracted the data and evaluated the study quality. We pooled the results using fixed effects models where statistically significant heterogeneity (p ≤ 0.1) was absent.

**Results:**

We identified six trials including 230 patients. Respiratory rehabilitation reduced the risk for hospital admissions (pooled relative risk 0.26 [0.12–0.54]) and mortality (0.45 [0.22–0.91]). Weighted mean differences on the Chronic Respiratory Questionnaire were 1.37 (95% CI 1.13–1.61) for the fatigue domain, 1.36 (0.94–1.77) for emotional function and 1.88 (1.67–2.09) for mastery. Weighted mean differences for the St. Georges Respiratory Questionnaire total score, impacts and activities domains were -11.1 (95% CI -17.1 to -5.2), -17.1 (95% CI -23.6 to -10.7) and -9.9 (95% CI -18.0 to -1.7). In all trials, rehabilitation improved exercise capacity (64–215 meters in six-minute walk tests and weighted mean difference for shuttle walk test 81 meter, 95% CI 48–115).

**Conclusion:**

Evidence from six trials suggests that respiratory rehabilitation is effective in COPD patients after acute exacerbation. Larger trials, however, are needed to further investigate the role of respiratory rehabilitation after acute exacerbation and its potential to reduce costs caused by COPD.

## Introduction

Acute exacerbations of chronic obstructive pulmonary disease (COPD) represent a major burden for patients and health care systems. For patients, acute exacerbations are a common reason for hospital admissions and severely affect health-related quality of life (HRQL) [1] and prognosis[2]. Mortality rates during hospitalisations are around 10% [3,4] and during the year following a hospitalisation may be as high as 40% [3,5].

From the health care provider's perspective, COPD is resource consuming [6]. A small proportion of COPD patients of around 10% suffering from acute exacerbations accounts for over 70 percent of costs caused by COPD because of emergency visits and hospitalisations [6-8]. The readmission rate is typically high in these high-risk patients. A recent large study found a readmission rate of 63% during a mean follow-up of 1.1 year with physical inactivity amongst the significant predictors for readmissions[9].

Recent position papers of the American College of Physicians and American College of Chest Physicians provided recommendations on the management of acute exacerbations [10,11]. However, a weakness of these papers was that they did not provide recommendations how future exacerbations and hospitalisations could be prevented despite being one of the main goals of COPD management [11,12]. One solution that has been adopted in clinical practice is to provide rehabilitative care after treatment of acute exacerbation including physical exercise, patient education focusing on self-management strategies and psychosocial support. The rationale to offer rehabilitation in patients recently treated for acute exacerbation is to enhance HRQL as in stable COPD patients [13], but also to modify factors associated with increased risk for post-exacerbation morbidity and mortality. A recent study showed that exacerbations results in acute muscle deconditioning and weakness[14]. Hence patients with frequent exacerbations have more pronounced skeletal muscle weakness and a more limited six minute walking distance [15], which is in turn a risk factor for exacerbations and mortality [3,16].

Thus respiratory rehabilitation may have the potential to reduce hospital admissions by improving exercise capacity. It is hence surprising, and in contrast to the large body of evidence supporting respiratory rehabilitation in stable patients [13,17], that the effects of respiratory rehabilitation in patients after acute exacerbation has never been studied systematically. Therefore, our aim was to conduct a systematic review of all randomized controlled trials that compared respiratory rehabilitation after acute exacerbation and usual care.

## Methods

### Identification of studies

We used five strategies to identify studies including electronic databases, consultations with experts from North America and Europe, our own files, bibliographies of articles that met the inclusion criteria and conference proceedings of the International Conference of the American Thoracic Society and the Congress of the European Respiratory Society.

An information specialist conducted electronic database searches in MEDLINE (Ovid version, New York, New York, from inception to April 2005), EMBASE (DataStar version, Cary, North Carolina from inception to April 2005), PEDRO (online version, University of Sydney, Australia, April 2005) and the Cochrane Central Register of Controlled Trials (Oxford, United Kingdom, 2005, Issue 1). We did not restrict the search to COPD patients with exacerbation only because exacerbation is not indexed as a Medical subject heading term and we feared to miss relevant studies with a narrow search. We used a broad search strategy using the terms "lung diseases obstructive", "chronic obstructive lung disease", "chronic obstructive pulmonary disease", "rehabilitation", "exercise", "exercise movement techniques", "physical endurance", "muscle training", "kinesiotherapy", "clinical trial", "controlled study" and "epidemiologic methods". A detailed search strategy is available on request. We also searched the Science Citation Index database (Web of Science, Thomson ISI, Philadelphia, Pennsylvania) and the "related articles" function of PubMed (National Library of Medicine, 8600 Rockville Pike, Bethesda, MD 20894) by entering all included studies.

### Inclusion criteria

We included randomized controlled trials comparing respiratory rehabilitation of any duration after acute exacerbation of COPD with conventional care. Respiratory rehabilitation programmes needed to include at least physical exercise. We included studies if more than 90% of study participants had COPD. Main outcome measure was unplanned hospital admissions and secondary outcomes included exacerbations, outpatient visits, dyspnea, HRQL as measured by disease-specific or generic questionnaires, functional and maximum exercise capacity, mortality and adverse events during rehabilitation. We did not apply any language restrictions.

### Study selection

**Figure 1:**
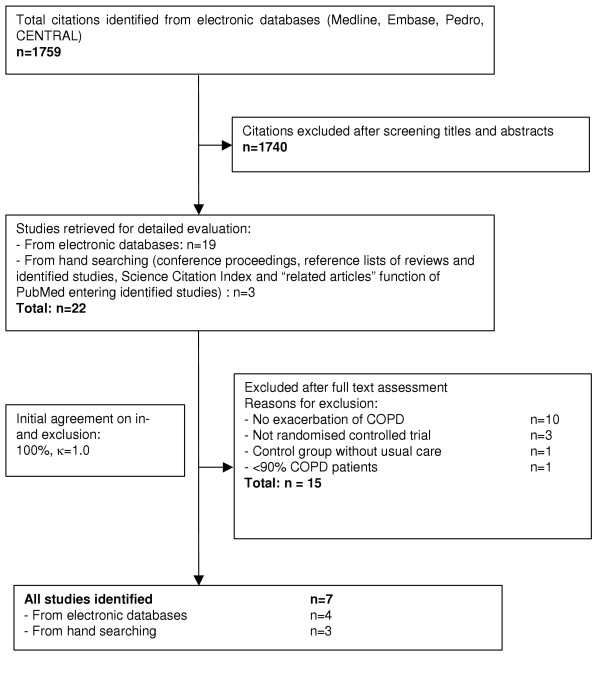
Study flow from identification to final inclusion of studies.

The bibliographic details of all retrieved articles were stored in a Reference Manager file (Professional Edition Version 10, ISI ResearchSoft, Berkeley, California). We removed duplicate records resulting from the various database searches. Two members of the review team (MAP, MS) independently scrutinized the titles and abstracts of all identified citations (see Figure 1) and ordered the full text of any article that was deemed potentially eligible by one of the reviewers. The two reviewers evaluated the full text of all retrieved papers, made a decision on in- or exclusion and discussed the decisions. Any disagreement was resolved by consensus with close attention to the inclusion/exclusion criteria. We recorded the initial degree of discordance between the reviewers and corrected discordant scores based on obvious errors. We resolved discordant scores based on real differences in interpretation through consensus or third party arbitration.

### Data extraction and quality assessment

We performed the data extraction using pilot-tested data forms. One reviewer extracted details about study patients, interventions and outcome measures as well as the results in a predefined data form and the second reviewer checked the data extraction for accuracy. We contacted all authors of the primary studies to obtain missing information. Two reviewers independently evaluated the quality of included trials using a detailed list of quality items assessing components of internal validity (Table 2) [18]. We did not rate the two items "blinding of patients" and "blinding of persons who implements intervention" because patients and treatment providers cannot be blinded in studies comparing respiratory rehabilitation and usual care.

**Table 2:** Quality assessment

Study	Prognostically homogenous study population	Concealment of random allocation	Prestratification on prognostically relevant variables	Description of randomisation procedure	Registration of loss to follow-up	Registration of co-interventions for each group	Blinding of outcome assessors	Check success of blinding
Behnke [19, 20]	+/-	-	-	-	+	+/-	-	-
Kirsten 1998 [22]	+/-	-	-	-	+	+/-	-	-
Man 2004 [24]	+/-	+	+	+/-	+	-	-	-
Murphy 2005 [21]	+/-	+	-	-	+	-	-	-
Nava 1998 [23]	+/-	-	-	+/-	+	-	-	-
Troosters [25, 26]	+/-	+	-	-	+	-	-	-

+: Fulfilled; +/-: Partially fulfilled; -: Not fulfilled or no information provided

### Methods of analysis and synthesis

We summarized the results of the data extraction and assessment of study validity in structured tables. We pooled trial results using fixed effects models if there was no significant heterogeneity (p ≤ 0.1 with Q statistic for continuous and Cochran chi-squared test for binary outcomes). In anticipation of significant heterogeneity we established a priori hypotheses to explain differences in outcomes across studies. First, heterogeneity may arise from the setting patients were recruited (in- or outpatient treatment of exacerbation), second from different lengths of follow-up, third from different length of the intervention and finally from differences in the methodology of the intervention. Pooled risk ratios and 95% confidence intervals (CIs) were computed by calculating weighted mean differences and pooled risk ratios using STATA (version 8.2, Stata Corp., College Station, Texas).

## Results

We show the study selection process and agreement on study inclusion in Figure 1. Out of the 22 potentially relevant articles, we included seven reports (Table 1). Two articles were based on the same trial. One reported the results after six [19] and the other one after 18 months [20]. In five trials, patients were recruited after inpatient care and in one trial [21] after hospital at home treatment for acute exacerbation. Two trials reported on the short-term benefit of inpatient rehabilitation programs [22,23] and four trials had rehabilitation programs of six weeks to six months duration [20,21,24,25]. One trial was published as an abstract only [25], but additional information was available from an earlier publication[26] and from the author. Altogether 140 patients were randomized to the rehabilitation intervention, and 90 were randomized into respective control groups.

**Table 1:** Characteristics of included studies

**Study**	**Population**	**Intervention**	**Follow up**	**Outcomes**
Behnke 2000 [19] and 2003 [20]	26 COPD patients (mean age 67 years, 77% males, mean FEV_1 _= 36% predicted) after inpatient treatment for acute exacerbation.	**Rehabilitation: **Within 4–7 days after admission, inpatient respiratory rehabilitation with endurance exercise (5 walking sessions/day for 10 days), followed by six months of supervised home-based endurance exercise (3 walking sessions/day for 6 months) **Usual care: **Standard inpatient care without exercise and standard community care with respirologist.	18 months	CRQ, Transition dyspnea index, 6 MWT, hospital readmission, mortality
Kirsten 1998 [22]	29 COPD patients (mean age 64 years, 90% males, mean FEV_1 _= 36% predicted) after inpatient treatment for acute exacerbation.	**Rehabilitation: **Within 6–8 days after admission, inpatient respiratory rehabilitation with endurance exercise (5 walking sessions/day for 10 days). **Usual care: **Standard inpatient care without exercise.	11 days	Transition dyspnea index, 6 MWT
Man 2003 [24]	42 COPD patients (mean age 70 years, 41% males, FEV_1 _= 39% predicted) after inpatient treatment for acute exacerbation.	**Rehabilitation: **Multidisciplinary outpatient respiratory rehabilitation (within 10 days of discharge) with endurance and strength exercise and patient education for 12 weeks (2 sessions/week). **Usual care: **Standard community care with respirologist	12 weeks	CRQ, SGRQ, Short form survey 36, shuttle walk test, hospital readmission, hospital days, emergency admissions, mortality
Murphy 2005 [21]	26 COPD patients (mean age 66 years, 65% males, mean FEV_1 _= 40% predicted) after home for hospital treatment for acute exacerbation.	**Rehabilitation: **Supervised home-based respiratory rehabilitation with endurance and strength exercise for 6 weeks (2 supervised sessions/week and daily unsupervised sessions). **Usual care: **Standard community care with respirologist	6 months	SGRQ, EuroQol, MRC dyspnea scale, shuttle walk test, 3-minute step test, hospital readmission
Nava 1997 [23]	70 COPD patients (mean age 66 years, 73% males, mean FEV_1 _= 32% predicted, 76% needed mechanical ventilation) admitted to inpatient care for treatment of acute exacerbation.	**Rehabilitation**: Within 3–5 days after admission, inpatient respiratory rehabilitation with four steps of increasing intensity. Step I, if unable to walk: Mobilisation and strength training for lower extremities. Step II, if able to walk: Endurance exercise (walking) Step III, if possible: Endurance exercise (cycling and stair climbing) and respiratory muscle training IV, if possible: Endurance exercise (cycling at highest tolerated intensity, 2 sessions/day for 3 weeks) **Usual care: **Only steps I and II.	6 weeks	Dyspnea on exertion, 6 MWT, mortality
Troosters 2002 [25, 26]	48 COPD patients (mean age 62 years, 85% males, FEV_1 _= 39% predicted) after inpatient treatment for acute exacerbation.	**Rehabilitation: **Outpatient respiratory rehabilitation with endurance and strength exercise for 6 months (3 sessions/week in first 3 months, then 2/week). **Usual care: **Standard community care with respirologist.	6 months (6 MWT) and 4 years (survival)	6 MWT, mortality

6-MWT: 6-minute walk test; CRQ: Chronic Respiratory Questionnaire; SGRQ: St. Georges Respiratory questionnaire; MRC: Medical Research Council

Initial agreement of reviewers on quality assessment was 85% for all items (chance corrected kappa = 0.70). All disagreement could be resolved by consensus. The quality of trials was moderate (Table 2). Three trials provided details about the randomisation procedures and three trials about concealment of random allocation, while in none of the trials the outcome assessors were blinded.

### Effect on hospital admissions

**Figure 2:**
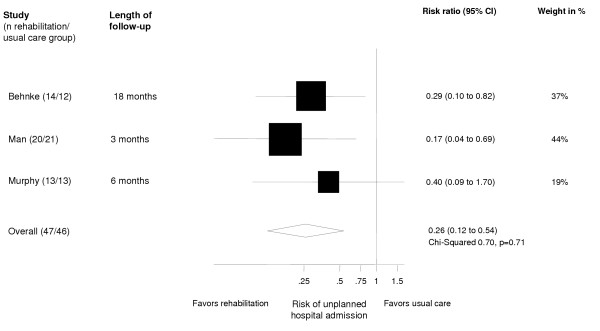
Effect of respiratory rehabilitation on unplanned hospital admissions. Boxes with 95% confidence intervals represent point estimates for the risk ratio.

Figure 2 shows the effect of respiratory rehabilitation on unplanned hospital admissions for each study [20,21,24] and the pooled relative risk ratio of 0.26 (0.12–0.54). The other trials included either only inpatients [22,23] or did not record hospital admissions during the follow-up [25].

### Effect on HRQL

**Figure 3:**
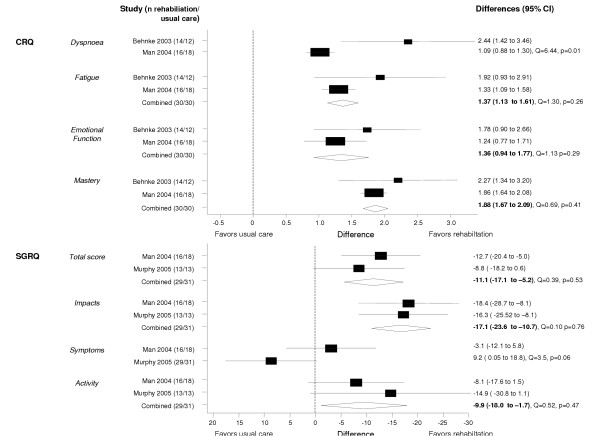
Effect of respiratory rehabilitation on Health-related quality of life as assessed by the Chronic Respiratory Questionnaire (CRQ) and St. Georges Respiratory Questionnaire (SGRQ). Boxes with 95% confidence intervals represent point estimates for the difference between respiratory rehabilitation and usual care.

Three trials assessed HRQL using the Chronic Respiratory Questionnaire (CRQ) [20,24] and the St Georges Respiratory Questionnaire (SGRQ) [21,24] (Figure 3). With both instruments, the trials found large effects exceeding the minimal important difference of 0.5 on the CRQ and of 4 on the SGRQ. Weighted mean differences (expressed as points change on a scale from 1 to 7) on the CRQ were 1.37 (95% CI 1.13–1.61) for the fatigue domain, 1.36 (0.94–1.77) for emotional function and 1.88 (1.67–2.09) for mastery. Weighted mean differences for the SGRQ total score, impacts and activities domains were -11.1 (95% CI -17.1 to -5.2), -17.1 (95% CI -23.6 to -10.7) and -9.9 (95% CI -18.0 to -1.7). For the CRQ dyspnea and SGRQ symptoms domain, results were too heterogeneous to be pooled (Q = 6.44, p = 0.01 for CRQ dyspnea domain and Q = 3.50, p = 0.06 for SGRQ symptoms domain), but all studies showed a consistent effect in favor of the rehabilitation intervention.

Man and Murphy also used generic HRQL instruments and found improvements by respiratory rehabilitation of 10.6 (-0.3 to 21.6) and 20.1 (3.3 to 36.8) on the physical composite and mental composite score of the Short-Form Survey 36 [24] and of 0.18 (95% CI 0.04 to 0.32) with the EuroQol score [21].

### Effect on dyspnea

In the trial by Behnke [20], the mean difference between groups on the transition dyspnea index was 6.9 (3.9 to 9.9) at the end of the treatment period and 8.6 (6.3–10.9) after 18 months. Kirsten[22] found significant differences in Transition dyspnea index scores after a short inpatient rehabilitation (p < 0.05, no additional data available) and Nava [23] also observed a significant effect of rehabilitation on dyspnoea (difference between groups 17 mm on visual analogue scale after a 50 meter walk, p < 0.01). Murphy [21] used the Medical Research Council dyspnea scale and also found that respiratory rehabilitation decreased dyspnea by 0.3 although this did not reach statistical significance (95% CI -0.92 to 0.32).

### Effect on exercise capacity

**Figure 4:**
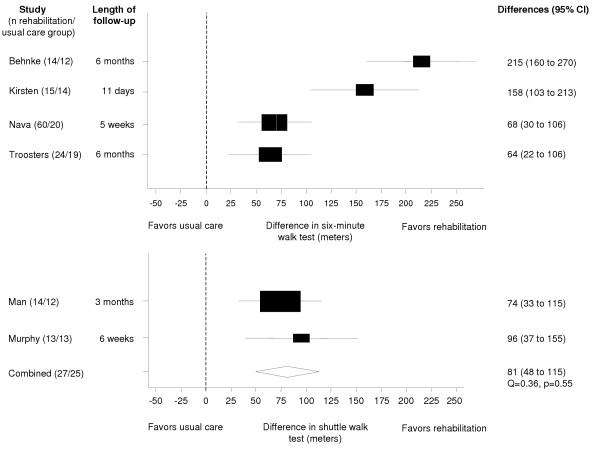
Effect of respiratory rehabilitation on six-minute walking and shuttle walk distance. Boxes with 95% confidence intervals represent point estimates for the difference between respiratory rehabilitation and usual care.

All trials showed a significant benefit of respiratory rehabilitation on the six-minute walking distance (Figure 4). We did not pool the results of the six-minute walking tests because of statistically significant heterogeneity (Q = 28.33, p < 0.001), which could not be explained by our a priori defined sources for heterogeneity. The trials reported by Behnke [19] and Kirsten[22] were conducted in the same institution and showed much larger effects (mean effects of 215 and 158 meters on the six minute walking test) compared to the trials of Nava [23] (68 meters) and Troosters [25] (64 meters). All studies showed a consistent benefit in favor of the rehabilitation group, which exceeded the minimal clinically important difference of 53 meters. The meta-analysis of the shuttle walk tests results showed a weighted mean difference of 81 meters (95% CI 48 to 115) between the rehabilitation and usual care groups.

### Effect on mortality

**Figure 5:**
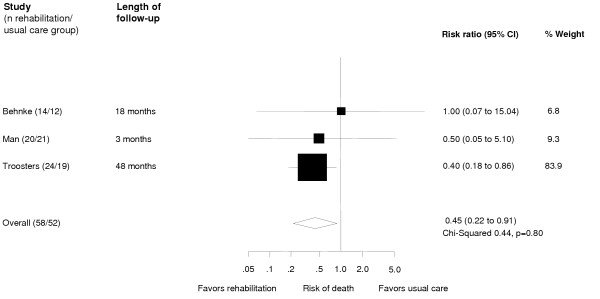
Effect of respiratory rehabilitation on mortality. Boxes with 95% confidence intervals represent point estimates for the risk ratio.

The individual study relative risks for mortality ranged from 0.40 (0.18–0.86) to 1.00 (0.07–15.04, Figure 5). The pooled risk ratio was 0.45 (0.22–0.91). Although no significant heterogeneity was present, it should be noted that the length of follow-up differed substantially between these studies. We did not include one trial [23] in the primary meta-analysis because severity of disease of included patients differed considerably from those of the other studies. For this trial a mortality of 20% for patients of either group (12/60 in rehabilitation group and 4/20 in control group) was observed while staying in the respiratory intensive care unit with a mean survival of 18.1 days (SD 7.2) for patients with and 12.4 days (SD 11.1) for patients without rehabilitation (p > 0.05). Of the 12 patients of the rehabilitation group who died, only five started a walking training (stage 2, Table 1). If this trial is included in the meta-analysis the pooled risk ratio is 0.59 (0.34–1.05) favoring the rehabilitation group.

### Adverse events

Two trials explicitly recorded adverse events. Neither Man [24] nor Behnke [19] observed adverse events during the rehabilitation.

## Discussion

The meta-analyses showed that respiratory rehabilitation after acute exacerbation of COPD reduced the risk for hospital admissions and mortality and led to large improvements of HRQL and exercise capacity.

Strengths of this systematic review include the extensive literature search, rigorous adherence to a predefined protocol and contacts to authors of the included trials who all provided additional information about their data. A limitation is the small number of patients included in the trials and methodological shortcomings that limit conclusions.

The effect of respiratory rehabilitation after acute exacerbation appears to be large. For HRQL and exercise capacity, the effects were well above the threshold for the minimal important difference for the CRQ (0.5 point difference [27]), St. Georges Respiratory Questionnaire (4 points [28]), SF-36 (5 points[29]) and Six-minute walking distance (around 53 meters [30]). In addition, the number of unplanned hospital admissions and mortality was reduced substantially. When one assumes that respiratory rehabilitation improves activity level in patients with COPD, it seems plausible that rehabilitation reduces readmission rate as inactivity has been shown to be a predictor of readmissions[9].

Compared to respiratory rehabilitation in stable COPD patients [13], its effects tend to be even larger after acute exacerbation. Several factors may contribute to this. First, as mentioned above, exacerbations lead to significant reductions in muscle function[14] and quality of life [1]. This initial deterioration may render patients more likely to improve from respiratory rehabilitation. Second, since patients were hospitalized, there may be a deficiency in self-management, or education. This may be partially targeted with the rehabilitation intervention, and patient education, as an additional part of multidisciplinary rehabilitation programs, may be of particular benefit to modify behavior. Indeed, a recent study showed impressive results of a patient management program including home exercises for COPD patients after acute exacerbation [31]. The mean number of hospital admissions per patient was reduced from 1.6 to 0.9 in the year following a hospital admission due to acute exacerbation. It is well known from earlier studies that the recovery period is long even in patients who have no further exacerbations and that another exacerbation within 6 months limits recovery markedly [32]. Our meta-analyses showed that respiratory rehabilitation during the recovery period is superior compared with usual care to improve prognosis and HRQL.

A word of caution is needed when interpreting the current analysis. A clear limitation of the trials is their relatively small sample size. All trials, in particular the trials reported by Behnke [20] and Kirsten[22] showed large effects of respiratory rehabilitation on HRQL and exercise capacity. Small trials tend to overestimate the effect of an intervention compared to large trials [33-36]. This phenomenon can partly be attributed to a publication bias, that is, the fact that small trials are more likely to be published if they show statistically significant treatment effects [37]. On the other hand, methodological shortcomings of small trials such as inadequate generation of the randomisation code, insufficient concealment of random allocation and lack of blinding contribute to discrepancies between the results of single large trials and pooled estimates based on small trials[35]. In our systematic review, the trials had methodological limitations and it cannot be excluded that the estimates provided by the meta-analyses represent overestimations of the effect of respiratory rehabilitation after acute exacerbation.

Larger trials seem justified to challenge the data presented in this article. Such trials should assess the effect of respiratory rehabilitation on unplanned out- and inpatient care but also include data on patient-important outcomes such as HRQL. Conducting trials on respiratory rehabilitation after acute exacerbation is, however, challenging. First, recruitment of patients is difficult because not all of them may want to be randomly allocated to respiratory rehabilitation or usual care in a situation of poor health status. Second, one needs to take into consideration that exercise capacity is particularly low after acute exacerbations[14] so that the exercise program should be designed carefully. Strength exercise and tolerable whole body exercise modalities such as interval exercise may be particularly suitable for these patients [38,39]. Third, the definition of usual care raises a number of difficulties. Patients willing to participate in the trial are likely to have a preference for respiratory rehabilitation. If they are randomized to the control group, they might ask for respiratory rehabilitation at any time during the follow-up. Given the clear benefits of this intervention in stable patients, confirmed in meta-analyses [13], patients should not be refrained from rehabilitative strategies. It would perhaps be ethically justifiable to conduct a large rehabilitation trial in places where respiratory rehabilitation is currently not readily available to the general patient. This appears to be the case in many countries including Switzerland [40], the UK [41] and Canada [42]. These countries are just few examples of countries where the lack of access to rehabilitation has been pointed out as an important caveat in health care. In these places patients could be randomized to additional respiratory rehabilitation or standard treatment by general practitioners and respirologists because respiratory rehabilitation can be offered to a small proportion of COPD patients only. Alternatively relatively short term studies (3–6 months follow-up) could be conducted with re-admission as a primary end point. It has been shown that re-admission occurs often soon after discharge [43,44]. Obviously, such studies could never address mortality as a primary end point, due to a lack of events. Whatever design investigators choose, a careful discussion of ethical and methodological issues is necessary before conducting large trials.

The present data show that respiratory rehabilitation has the potential to reduce the large COPD-related costs due to hospital admissions. It may not only reduce the number of acute exacerbations but also their severity. Patients may learn to notice imminent exacerbations and seek medical attention earlier leading to a shift from inpatient to the less costly outpatient treatment of acute exacerbations. The significant reduction in hospital readmissions is suggestive of a beneficial cost-benefit balance. However, larger trials should provide the final evidence base for formal cost analyses to test the hypothesis that respiratory rehabilitation after acute exacerbation is cost effective.

The data presented in this review are the first to show a survival benefit of respiratory rehabilitation in patients at risk. Although the results should be interpreted with caution, as mentioned above, this study provides the most solid evidence currently available that mortality is reduced. In summary, current evidence suggests that respiratory rehabilitation reduces unplanned hospital admissions and mortality and improves HRQL and exercise capacity when initiated immediately after acute exacerbations.

## Abbreviations

COPD: Chronic obstructive pulmonary disease

HRQL: Health-related quality of life

CI: confidence interval

CRQ: Chronic Respiratory Questionnaire

SD: Standard deviation

## Contributions

Protocol writing: Puhan, Scharplatz, Steurer

Acquisition of data: Puhan, Scharplatz

Analysis and interpretation of data: Puhan, Scharplatz, Troosters, Steurer

Drafting of manuscript: Puhan

Critical revision of manuscript for important intellectual content: Puhan, Scharplatz, Troosters, Steurer

## Conflict of interest

The author(s) declare that they have no competing interests.

## Funding

Helmut Horten Foundation; Zurich Lung League. Thierry Troosters is a postdoctoral fellow of the Fonds voor Wetenschappelijk Onderzoek-Vlaanderen.
